# Predictors for trifecta achievement of robot‐assisted partial nephrectomy in high‐complexity tumors (Preoperative Aspects and Dimensions Used for an Anatomical score ≥10)

**DOI:** 10.1111/ases.12767

**Published:** 2019-12-10

**Authors:** Kiyoshi Takahara, Makoto Sumitomo, Kosuke Fukaya, Takahito Jyoudai, Masashi Nishino, Masaru Hikichi, Takuhisa Nukaya, Kenji Zennami, Manabu Ichino, Naohiko Fukami, Hitomi Sasaki, Mamoru Kusaka, Ryoichi Shiroki

**Affiliations:** ^1^ Department of Urology Fujita Health University School of Medicine Toyoake Japan

**Keywords:** high‐complexity tumor, PADUA score, RAPN

## Abstract

**Introduction:**

Robot‐assisted partial nephrectomy (RAPN) is emerging as an effective treatment oncologically and functionally for clinically localized renal tumors. However, RAPN in high‐complexity tumors with a Preoperative Aspects and Dimensions Used for an Anatomical score ≥10 remains challenging. In this study, the feasibility of RAPN for high‐complexity tumors was assessed.

**Methods:**

The study cohort consisted of 177 cases with clinically localized renal cell carcinoma who had undergone RAPN at our hospital from July 2010 to February 2018. They were assessed for perioperative parameters and trifecta achievement (ie, negative surgical margins, warm ischemia time <25 minutes, and no complications).

**Results:**

Among the 177 cases who had undergone RAPN, 60 had high‐complexity tumors, and 117 had non‐high‐complexity (ie, intermediate‐ or low‐complexity) tumors. There were no significant differences in the operative and console times between the cohorts, but estimated intraoperative blood loss was much lower in the non‐high‐complexity group. Although the average warm ischemia time was less than 25 minutes in both groups, it was significantly shorter in the non‐high‐complexity group. Trifecta achievement rates significantly differed between the high‐ and non‐high‐complexity groups (68.3% vs 86.3%). Comparisons of four operative parameters (ie, BMI, tumor size, endophytic properties, and hilar tumor) using univariate analysis in the 60 high‐complexity tumor cases showed that BMI and tumor size were independent factors (*P* = 0.05 and 0.018, respectively). In multivariate analysis, tumor size was the only factor directly associated with trifecta achievement (*P* = 0.029).

**Conclusion:**

The trifecta achievement rate was significantly lower in the high‐complexity group. Only tumor size affected trifecta achievement during RAPN in cases with high‐complexity tumors (Preoperative Aspects and Dimensions Used for an Anatomical score ≥10).

## 1 INTRODUCTION

Renal cell carcinoma was projected to be associated with approximately 63 990 newly diagnosed kidney cancer cases and 14 400 cancer‐related deaths in 2017 in the United States.^1^ Compared to radical nephrectomy (RN), partial nephrectomy (PN) provides better outcomes with regard to surgery‐related mortality, cancer‐specific survival, time‐to‐recurrence, and renal function for a cT1a renal mass and has been established as a standard management protocol.^2^, ^3^ Moreover, in the management of larger tumors (cT1b and cT2), a recent review suggested that PN was a viable treatment option because it offered acceptable surgical morbidity and, compared to RN, equivalent cancer control, better preservation of renal function, and potential for better long‐term survival.^4^ PN can be performed using a robot‐assisted, laparoscopic, or open approach. Since its introduction, robot‐assisted partial nephrectomy (RAPN) has been shown to be a feasible alternative to open PN^5^ and to provide similar or better perioperative outcomes than laparoscopic PN.^6^, ^7^, ^8^

Some recently developed standardized anatomical classification scoring systems categorize and stratify patients into different anatomical complexity groups and allow urologists to estimate the potential perioperative outcomes.^9^, ^10^, ^11^, ^12^, ^13^, ^14^ Among them, the R.E.N.A.L. (radius [tumor size as maximal diameter], exophytic/endophytic properties of the tumor, nearness of the deepest portion of the tumor to the collecting system or sinus, anterior/posterior descriptor, and the location relative to the polar line) nephrometry score and Preoperative Aspects and Dimensions Used for an Anatomical (PADUA) score have been most frequently used.^9^, ^10^

A composite outcome metric, the trifecta (ie, a negative surgical margin, warm ischemia time <25 minutes, and no complications), has been suggested as a measure of operative quality after PN.^15^, ^16^, ^17^ In this study, we hypothesized that some perioperative parameters (ie, BMI, tumor size, endophytic properties, and hilar tumor) might be associated with the trifecta achievement of RAPN for high‐complexity tumors with a PADUA score ≥10 and validated in multivariate analysis.

## 2 METHODS

### 2.1 Study design

This study was an analysis of 177 cases who had undergone RAPN at our hospital from July 2010 to February 2018. Operative variables including age, gender, BMI, tumor side, operation approach (transperitoneal or retroperitoneal), R.E.N.A.L. nephrometry score, and PADUA score were extracted. Before surgery, all patients underwent 3‐D CT or MRI to define tumors’ clinical stage and anatomical characteristics. Patients were divided into two groups according to PADUA score: the high‐complexity group (PADUA score ≥10) and the non‐high‐complexity group (ie, intermediate‐ or low‐complexity group) (PADUA score 6‐9). To assess perioperative parameters, operative time, console time, estimated blood loss (EBL), and warm ischemia time (WIT) were checked. Trifecta achievement was a composite outcome measure for assessing quality of surgery in RAPN that consists of a WIT ≤25 minutes, no complications, and a negative surgical margin. Complications were defined as those that were Clavien‐Dindo ≥grade III.

To perform RAPN, tumor depth was assessed with a laparoscopic ultrasound. After the administration of mannitol, the renal artery or its branches were clamped with a bulldog clamp. The tumor was resected with 2‐5 mm of the parenchymal margin. For the inner renorrhaphy layer, the collecting system and large vessels were closed with 3‐0 V‐Loc sutures, and if needed, parenchymal sutures were made with 2‐0 V‐Loc. Seven surgeons who completed the da Vinci certification program approved in Japan performed RAPN. We performed RAPN for the first time on July 29, 2010.

The protocol for this study was approved by our institution's ethics committee (approval no. HM 16‐340), and the study was performed in accordance with the ethical standards established in the most recent version of the Declaration of Helsinki.

### 2.2 Statistical analysis

All values are presented as means ± SD, and statistical comparisons of the results were performed with Student's *t* test, Mann‐Whitney *U* test, χ^2^ test, or Fisher's exact test. To assess independent prognostic factors for trifecta achievement in the high‐complexity group, univariate analysis was performed with BMI, size, endophytic properties, and hilar tumor as variables. Significant preoperative variables in the univariate analysis were included in multivariate analysis using a Cox proportional hazards regression model. In all statistical analyzes, *P* < .05 was considered significant. All data were analyzed using IBM SPSS version 23 (SPSS Japan Inc., Tokyo, Japan).

## 3 RESULTS

### 3.1 Clinical characteristics of patients

The study cohort consisted of 177 cases with clinically localized renal cell carcinoma who had undergone RAPN at our hospital from July 2010 to February 2018. There were 60 cases in the high‐complexity group (high cohort) and 117 cases in the non‐high‐complexity group (non‐high cohort) (Table 1). The mean age and BMI were 59.5 years and 23.6 kg/m^2^ in the high cohort and 58.7 years and 23.7 kg/m^2^ in the non‐high cohort. The transperitoneal approach was used more than the retroperitoneal approach in both groups. The mean R.E.N.A.L. nephrometry score and PADUA score were 8.4 and 10.8 in the high cohort and 6.0 and 7.5 in the non‐high cohort. Among the factors age, gender, BMI, tumor side, approach, R.E.N.A.L. nephrometry score, and PADUA score, only the scores significantly differed between the two cohorts (*P* < .001).

**Table 1:** Clinical characteristics of patients

Baseline patient characteristics	High‐complexity group (n = 60)	Non‐high‐complexity group (n = 117)	*P*‐value
Mean age (y)	59.5	58.7	ns
Gender (n)			
Male	48	88	ns
Female	12	29	
Mean BMI (kg/m^2^)	23.6	23.7	ns
Tumor side (n)			
Right	30	57	ns
Left	30	60	
Approach			
Transperitoneal	34	66	ns
Retroperitoneal	26	51	
Mean R.E.N.A.L. nephrometry score	8.4	6.0	<.001
Mean PADUA score	10.8	7.5	

Abbreviations: ns, not significant; PADUA, Preoperative Aspects and Dimensions Used for an Anatomical; R.E.N.A.L., radius [tumor size as maximal diameter], exophytic/endophytic properties of the tumor, nearness of the deepest portion of the tumor to the collecting system or sinus, anterior/posterior descriptor, and the location relative to the polar line.

### 3.2 Perioperative parameters

**Figure 1:**
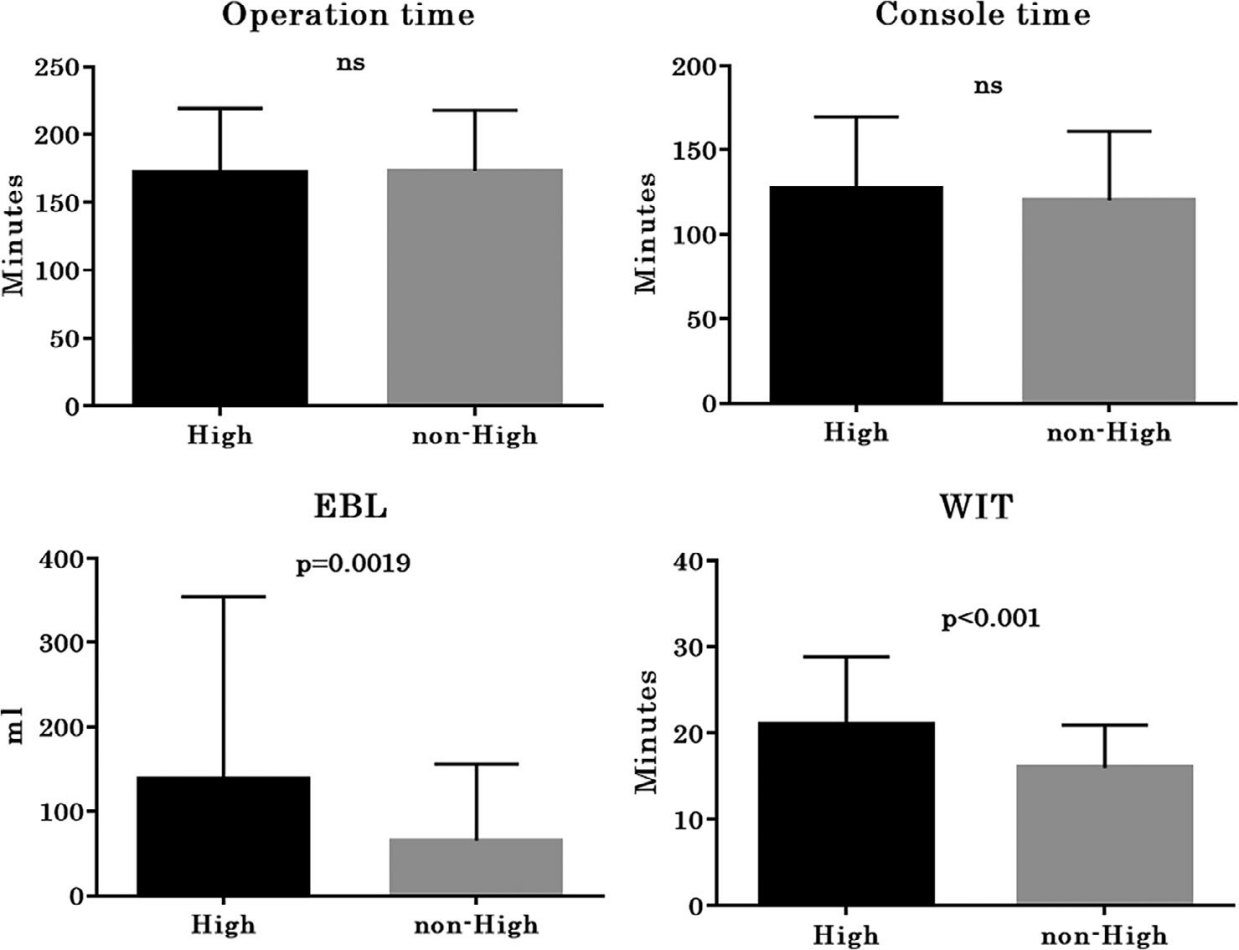
Perioperative parameters. EBL, estimated blood loss; WIT, warm ischemia time

Perioperative parameters including operative time, console time, EBL, and WIT are shown in Figure 1. The mean operative time and console time were 172 minutes and 127 minutes in the high cohort and 173 minutes and 120 minutes in the non‐high cohort; there were no significant differences. The mean EBL significantly differed between the high cohort and the non‐high cohort (138 vs 65 mL, *P* = .0019). Likewise, the mean WIT significantly differed between the high cohort and the non‐high cohort (21 vs 16 minutes, *P* < .001).

### 3.3 Trifecta achievement

We assessed trifecta achievement in both cohorts (Table 2). There was no case of positive surgical margins in either cohort. WIT <25 minutes was achieved in 44 cases (73.3%) in the high cohort and 110 cases (94.0%), representing a significant difference (*P* < .001). There were no complications classified as Clavien‐Dindo ≥grade III in 53 cases (88.3%) in the high cohort and 106 cases (90.6%) in the non‐high cohort; there was no significant difference between the groups. Complications included urine leak (high cohort, n = 3; non‐high cohort, n = 1), hemorrhage (high cohort, n = 4; non‐high cohort, n = 2), and others (non‐high cohort, n = 8). Overall, the trifecta was achieved in 41 cases (68.3%) in the high cohort and 101 cases (86.3%) in the non‐high cohort. There was significant difference in the rates of trifecta achievement between the cohorts (*P* = .009).

**Table 2:** Trifecta achievement

Baseline patient characteristics	High‐complexity group (n = 60), n (%)	Non‐high‐complexity group (n = 117), n (%)	*P*‐value
Negative surgical margins	60 (100)	117 (100)	ns
WIT <25 min	44 (73.3)	110 (94.0)	<.001
No complications	53 (88.3)	106 (90.6)	ns
Trifecta	41 (68.3)	101 (86.3)	0.009

Abbreviations: ns, not significant; WIT, warm ischemia time.

### 3.4 Cox regression analysis for trifecta achievement (high cohort)

**Figure 2:**
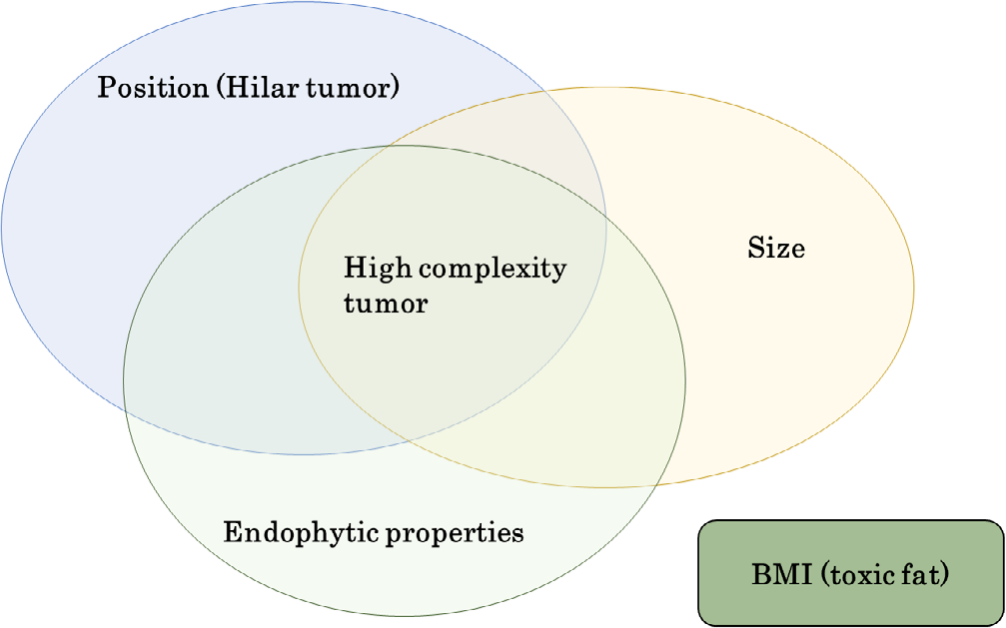
Factors preventing trifecta achievement in the high‐complexity group

We next evaluated which factors prevented trifecta achievement during RAPN in the high cohort. We focused on four factors: (a) tumor size, (b) endophytic properties, (c) hilar tumor, and (d) BMI. BMI was selected as a substitute for renal toxic fat (Figure 2). In univariate analysis in the high cohort, only tumor size was associated with WIT (*P* = .03), but in multivariate analysis, none of the four factors was associated with WIT (Table 3). Likewise, none of the four factors was independently associated with complications in multivariate analysis, but this was also true in univariate analysis (Table 4). When the four risk parameters in the high cohort were compared in multivariate analysis, tumor size remained associated with trifecta achievement (*P* = .029), but BMI did not (Table 5). These analyses demonstrate that tumor size was an important factor for trifecta achievement during RAPN in the high cohort.

**Table 3:** Cox regression analysis for warm ischemia time (high‐complexity group)

	Univariate analysis		Multivariate analysis	
	HR (95%CI)	*P*‐value	HR (95%CI)	*P*‐value
BMI (<25 vs >25 kg/m^2^)	0.482 (0.147‐1.585)	.229	0.604 (0.168‐2.168)	.44
Size (<4 vs >4 cm)	0.257 (0.076‐0.874)	.03	0.278 (0.073‐1.067)	.062
Endophytic properties (<50% vs >50%)	1.768 (0.488‐6.397)	.385	1.195 (0.268‐5.332)	.816
Hilar tumor (no vs yes)	1.714 (0.473‐6.212)	.412	1.735 (0.428‐7.031)	.44

Abbreviations: CI, confidence interval; HR, hazard ratio.

**Table 4:** Cox regression analysis for complications (high‐complexity group)

	Univariate analysis		Multivariate analysis	
	HR (95%CI)	*P*‐value	HR (95%CI)	*P*‐value
BMI (<25 vs >25 kg/m^2^)	1.181 (0.208‐6.715)	.852	1.656 (0.251‐10.908)	.6
Size (<4 vs >4 cm)	0.244 (0.048‐1.235)	.088	0.203 (0.033‐1.23)	.083
Endophytic properties (<50% vs >50%)	1.367 (0.235‐7.955)	.728	0.772 (0.107‐5.584)	.797
Hilar tumor (no vs yes)	1.286 (0.227‐7.293)	.777	1.502 (0.233‐9.675)	.669

Abbreviations: CI, confidence interval; HR, hazard ratio.

**Table 5:** Cox regression analysis for trifecta achievement (high‐complexity group)

	Univariate analysis		Multivariate analysis	
	HR (95%CI)	*P*‐value	HR (95%CI)	*P*‐value
BMI (<25 vs >25 kg/m^2^)	0.313 (0.097‐1.002)	.05	0.337 (0.099‐1.146)	.082
Size (<4 vs >4 cm)	0.235 (0.071‐0.783)	.018	0.251 (0.072‐0.086)	.029
Endophytic properties (<50% vs >50%)	0.914 (0.245‐3.418)	.894		
Hilar tumor (no vs yes)	1.444 (0.431‐4.84)	.551		

Abbreviations: CI, confidence interval; HR, hazard ratio.

## 4 DISCUSSION

In recent years, an increased number of cases have undergone RAPN because of the advantages on its minimal invasiveness compared to open and laparoscopic techniques. Also, RAPN has the advantages of a lower perioperative complication rate, shorter length of hospital stay, less EBL, shorter WIT, and better renal functional outcome than both open and laparoscopic PN.^18^, ^19^ However, the indications for RAPN for high‐complexity renal tumors have yet to be established because there has been very limited published evidence on RAPN's perioperative, oncological, and functional outcomes. In this study, the feasibility of RAPN was assessed, particularly for high‐complexity tumors with a PADUA score ≥10.

Regarding perioperative parameters, Abdel Raheem et al. reported that tumor complexity (ie, high, low, or medium) did not affect operative time.^20^ However, the average EBL increased with tumor complexity.^20^, ^21^ Our results regarding operative time and EBL are consistent with these previous studies.

Recent studies have demonstrated varying results with regard to trifecta achievement in high‐complexity tumors. Several have reported that high‐complexity tumors (PADUA score ≥10) could predict an increase in WIT.^22^, ^23^ In the present study, a significant difference was observed between the high cohort and non‐high cohort with regard to achieving WIT in less than 25 minutes. With regard to complications stemming from RAPN, Abdel Raheem et al. reported no difference between tumor complexity based on PADUA score and early postoperative complications according to the Clavien‐Dindo classification; the only difference among the groups divided by tumor complexity was rate of intraoperative conversion to RN, which was higher in the high‐complexity tumor group.^20^ Another recent paper reported that 22% cases with high‐complexity tumors were converted to RN, but no significant difference was observed in the early postoperative complication rate among the groups divided by tumor complexity.^21^ In the present study, no cases underwent open conversion, and there was no significant difference in the rate of Clavien‐Dindo ≥grade III complications between the high and non‐high cohorts. From a pathological perspective, Lista et al. reported that positive surgical margins were found in 6.5% of cases in their European Multicentre Observational Study (EMOS Project).^24^ In contrast, there were no cases of positive surgical margins in our present study.

Our study of trifecta achievement demonstrated that there was significant difference in PADUA score between the high cohort and non‐high cohort. This analysis of trifecta achievement in high‐complexity tumors is consistent with that in previous studies.^20^, ^25^ Some recent reports on predicting trifecta achievement indicated that ASA classification, operative time, and tumor size were independent factors.^20^, ^26^ In this study, we found that tumor size was an important factor for predicting trifecta achievement in patients with high‐complexity tumors undergoing RAPN. Because there have been very few papers on the factors that affect trifecta achievement, particularly in patients with high‐complexity tumors, this study could provide helpful guidance for surgeons trying to achieve the trifecta during RAPN in this patient group.

Our study encountered limitations as a result of the retrospective collection of data, the small sample from a single center, and the lack of well‐designed analyses. Therefore, further studies are required.

In conclusion, the trifecta achievement rate for high‐complexity tumors (PADUA score ≥10) was significantly lower than that for non‐high‐complexity tumors. Tumor size was the only important factor that affected trifecta achievement during RAPN for high‐complexity tumors.

## CONFLICT OF INTEREST

The authors have no potential conflicts of interest to disclose.
